# Outcomes and Predictors of Cure After Reoperation for Persistent and Recurrent Hyperparathyroidism: A Cohort Study in Saudi Arabia

**DOI:** 10.7759/cureus.91180

**Published:** 2025-08-28

**Authors:** Thikra M Alblowi

**Affiliations:** 1 Surgery, Yanbu General Hospital, Yanbu, SAU

**Keywords:** endocrine surgery, hyperparathyroidism, intraoperative parathyroid hormone monitoring, localization imaging, parathyroidectomy, persistent hyperparathyroidism, recurrent hyperparathyroidism, reoperation, saudi arabia

## Abstract

Background: Primary hyperparathyroidism is the leading cause of hypercalcemia in outpatients, and parathyroidectomy is the only curative treatment. Although initial surgery cures the majority of patients when performed by experienced surgeons, a subset of patients experience persistent or recurrent hyperparathyroidism, necessitating reoperation. Reoperation is technically demanding and associated with a higher complication risk, but it remains the only chance for a durable cure. Data on outcomes in the Middle East are scarce.

Methods: A retrospective cohort study of adults who underwent reoperation for persistent or recurrent hyperparathyroidism was conducted at tertiary centers in the Eastern Province of Saudi Arabia between 2008 and 2022. Persistent disease was defined as failure to normalize calcium and parathyroid hormone (PTH) within six months of index surgery, and recurrent disease was defined as return of hypercalcemia after at least six months of normocalcemia. Data on demographics, comorbidities, surgical and pathological features, imaging, and outcomes were collected. The primary outcome was biochemical cure at six months after reoperation. Secondary outcomes included complications, length of hospital stay, permanent hypoparathyroidism, late recurrence, and mortality. Predictors of cure were assessed using multivariable logistic regression.

Results: A total of 162 patients underwent reoperation (median age: 54 years; 114 (70.4%) women). Persistent hyperparathyroidism was present in 59 (36.4%) and recurrent disease in 103 (63.6%). Preoperative localization identified a lesion in 141 (87%). At reoperation, 71 (43.8%) underwent focused or minimally invasive approaches, and 76 (46.9%) underwent bilateral exploration. At six months, 131 (80.9%) achieved cure, including 42 of 59 (71.2%) with persistent disease and 89 of 103 (86.4%) with recurrent disease (p=0.02). Complications occurred in 21 (13%), including four (2.5%) with permanent recurrent laryngeal nerve injury and six (3.7%) with permanent hypoparathyroidism. On multivariable analysis, recurrent disease (adjusted odds ratio (OR): 2.4; 95% confidence interval (CI): 1.1-5.4), positive localization (OR: 3.1; 95% CI: 1.2-8.0), and adenoma pathology (OR: 2.8; 95% CI: 1.2-6.4) were independently associated with cure.

Conclusions: Reoperation for persistent or recurrent hyperparathyroidism was associated with durable cure in approximately four of five patients and acceptable morbidity. Cure was more likely in patients with recurrent disease, positive preoperative localization, and adenoma pathology, underscoring the importance of comprehensive imaging and specialized surgical expertise.

## Introduction

Primary hyperparathyroidism is the most common cause of hypercalcemia in outpatients, and parathyroidectomy remains the only curative therapy. When performed by experienced surgeons, initial parathyroidectomy achieves a cure in more than 95% of cases [1-3]. Nevertheless, a subset of patients experience either persistent hyperparathyroidism, defined as the failure to normalize calcium and parathyroid hormone (PTH) within six months of surgery, or recurrent hyperparathyroidism, defined as the return of hypercalcemia after a period of normocalcemia [3,4]. Together, these conditions occur in 2-10% of patients and represent one of the most challenging scenarios in endocrine surgery [5-7].

Reoperation for persistent or recurrent hyperparathyroidism is technically demanding. Distorted anatomy, scar tissue, and altered vascular supply increase the risk of complications, particularly recurrent laryngeal nerve (RLN) injury and permanent hypoparathyroidism [4-8]. Despite these challenges, successful reoperation offers the potential for a durable cure and relief of symptoms, particularly nephrolithiasis, bone disease, and neurocognitive complaints. Advances in preoperative localization, including 4D computed tomography and positron emission tomography with choline tracers, and the use of intraoperative PTH monitoring have improved surgical planning and outcomes. Still, success rates after reoperation are lower than those of initial parathyroidectomy, and outcomes vary across centers [5,9].

Data from the Middle East and Gulf region remain scarce, despite a growing burden of hyperparathyroidism related to high rates of chronic kidney disease and vitamin D deficiency. There is a need for regional evidence to guide patient selection, imaging strategies, and operative planning in reoperative cases. Accordingly, a retrospective study of patients undergoing reoperation for persistent or recurrent hyperparathyroidism was conducted in the Eastern Province of Saudi Arabia to characterize outcomes and identify predictors of cure.

## Materials and methods

Study design and setting

A retrospective cohort study of patients who underwent reoperation for persistent or recurrent hyperparathyroidism was conducted at tertiary referral centers in the Eastern Province of Saudi Arabia between January 2008 and December 2022. The study was approved by the Standing Committee for Sabbatical Leaves, Publication and Research Ethics of the Ministry of Health, Saudi Arabia (approval number: REC-2025-18107), and the requirement for informed consent was waived owing to the retrospective nature of the analysis.

Study population

Eligible patients were adults (≥18 years of age) who had undergone at least one prior parathyroidectomy for hyperparathyroidism and subsequently underwent reoperation for persistent or recurrent disease. Patients were excluded if the indication for reoperation was other than hyperparathyroidism (e.g., concomitant thyroid malignancy), if follow-up data within six months after reoperation were unavailable, or if operative notes were not retrievable. Persistent hyperparathyroidism was defined as hypercalcemia with elevated or inappropriately normal PTH within six months of the index surgery. Recurrent hyperparathyroidism was defined as the reappearance of hypercalcemia with elevated or inappropriately normal PTH after at least six months of documented normocalcemia.

Data collection

Data were abstracted from electronic and paper medical records using a standardized case report form. Collected variables included demographic characteristics, comorbidities, details of the index surgery (type of hyperparathyroidism, operative approach, intraoperative PTH monitoring, pathology, and complications), baseline and perioperative biochemical values, imaging studies performed before reoperation, and operative findings at reoperation. Postoperative outcomes were recorded, including complications, length of stay, biochemical cure at six months, late recurrence, and mortality. Cure was defined as normocalcemia with normal or appropriately suppressed PTH on at least two occasions six months or more after surgery. Permanent hypoparathyroidism was defined as a requirement for calcium and/or active vitamin D supplementation beyond six months, with documented hypocalcemia if supplementation was withheld.

Outcomes

The primary outcome was biochemical cure at six months after reoperation. Secondary outcomes included perioperative complications, length of hospital stay, persistent hypoparathyroidism, 30-day readmission, late recurrence of hyperparathyroidism, and mortality within 90 days of surgery.

Statistical analysis

Continuous variables are presented as means with standard deviations or medians with interquartile ranges, as appropriate, and categorical variables as frequencies and percentages. Between-group comparisons were made with the chi-squared test or Fisher's exact test for categorical variables and Student's t-test or the Wilcoxon rank-sum test for continuous variables. The association between clinical factors and cure at six months was evaluated using multivariable logistic regression, with covariates selected a priori on the basis of clinical relevance (disease type, localization status, operative approach, pathology, and intraoperative PTH monitoring). Results are presented as adjusted odds ratios with 95% confidence intervals. A two-sided p-value of less than 0.05 was considered statistically significant. Analyses were performed using Stata 17 (StataCorp LLC, College Station, Texas, United States).

## Results

Baseline characteristics

A total of 162 patients underwent reoperation for persistent or recurrent hyperparathyroidism. The median age was 54 years (interquartile range (IQR): 46-63), and 114 (70.4%) were women. The majority were Saudi nationals, 139 (85.8%). The mean body mass index was 28.7±4.9. Chronic kidney disease was present in 65 (40.1%), including 28 (17.3%) with stage 3, 21 (13%) with stage 4 or 5, and 16 (9.9%) on dialysis or post-transplant. A history of nephrolithiasis was documented in 61 (37.7%), fragility fracture in 29 (17.9%), and osteoporosis in 46 (28.4%). Diabetes mellitus was present in 57 (35.2%), and hypertension in 73 (45.1%) (Table 1).

**Table 1:** Baseline characteristics of the study population (n=162) MEN: multiple endocrine neoplasia; IQR: interquartile range

Characteristic		Overall (n=162)
Age, median (IQR), years		54 (46-63)
Female sex, no. (%)		114 (70.4)
Saudi national, no. (%)		139 (85.8)
Body mass index, mean±SD		28.7±4.9
Chronic kidney disease, no. (%)	None/stage 1-2	97 (59.9)
	Stage 3	28 (17.3)
	Stage 4-5	21 (13)
	Dialysis/post-transplant	16 (9.9)
MEN syndrome, no. (%)		9 (5.6)
History of nephrolithiasis, no. (%)		61 (37.7)
Fragility fracture, no. (%)		29 (17.9)
Osteoporosis diagnosis, no. (%)		46 (28.4)
Diabetes mellitus, no. (%)		57 (35.2)
Hypertension, no. (%)		73 (45.1)
Current or former smoker, no. (%)		41 (25.3)

Index surgery and early outcomes

At the time of initial surgery, 121 (74.7%) had primary hyperparathyroidism, 27 (16.7%) had secondary hyperparathyroidism, and 14 (8.6%) had tertiary hyperparathyroidism. Pathology revealed a single adenoma in 102 (63%), multigland hyperplasia in 41 (25.3%), double adenomas in nine (5.6%), and carcinoma in four (2.5%). Intraoperative PTH monitoring was used in 68 (42%). Complications occurred in 19 (11.7%), including transient RLN injury in five (3.1%), permanent RLN injury in two (1.2%), and permanent hypoparathyroidism in three (1.9%). The median length of stay was three days (IQR: 2-4). At six months, 133 (82.1%) achieved biochemical cure after the index surgery (Table 2).

**Table 2:** Index surgery and early outcomes (n=162) Shown are the types of hyperparathyroidism, histopathologic findings, operative details, and outcomes of the index surgery. Cure at six months was defined as normocalcemia with the appropriate suppression of PTH on two or more occasions. Complications are reported as transient or permanent. PTH: parathyroid hormone; RLN: recurrent laryngeal nerve; IQR: interquartile range

Variable		Overall (n=162)
Type of hyperparathyroidism, no. (%)	Primary	121 (74.7)
	Secondary	27 (16.7)
	Tertiary	14 (8.6)
Etiology at pathology, no. (%)	Adenoma	102 (63)
	Multigland hyperplasia	41 (25.3)
	Double adenoma	9 (5.6)
	Carcinoma	4 (2.5)
	Other/non-diagnostic	6 (3.7)
Intraoperative PTH monitoring used, no. (%)		68 (42)
Complications of index surgery, no. (%)	Any complication	19 (11.7)
	Transient RLN injury	5 (3.1)
	Permanent RLN injury	2 (1.2)
	Transient hypocalcemia	13 (8)
	Permanent hypoparathyroidism	3 (1.9)
Median length of stay (IQR), days		3 (2-4)
Cure at 6 months after index surgery, no. (%)		133 (82.1)

Characteristics of the reoperation episode

Of the reoperation cohort, 59 (36.4%) had persistent hyperparathyroidism, and 103 (63.6%) had recurrent disease. The median interval between the index surgery and reoperation was 24 months (IQR: 9-46). The mean pre-reoperation calcium level was 2.89±0.22 mmol/L, and the median PTH was 182 pg/mL (IQR: 134-254). Preoperative localization was successful in 141 (87%). Among individual modalities, ultrasound was positive in 104 (64.2%), sestamibi/single-photon emission computed tomography (SPECT-CT) in 86 (53.1%), 4D CT in 49 (30.2%), and positron emission tomography (PET) choline in 11 (6.8%) (Table 3).

**Table 3:** Characteristics of the reoperation episode (n=162) Shown are the indications for reoperation, time from index surgery, preoperative biochemical values, and results of localization imaging. Continuous variables are presented as mean±SD or median with IQR. Percentages are based on the total study population. PTH: parathyroid hormone; IQR: interquartile range; SPECT: single-photon emission computed tomography; PET: positron emission tomography

Variable		Overall (n=162)
Indication for reoperation, no. (%)	Persistent hyperparathyroidism	59 (36.4)
	Recurrent hyperparathyroidism	103 (63.6)
Interval, index to reoperation, median (IQR), months		24 (9-46)
Pre-reoperation calcium, mean±SD, mmol/L		2.89±0.22
Pre-reoperation PTH, median (IQR), pg/mL		182 (134-254)
Localization positive (any modality), no. (%)		141 (87)
Localization modality	Ultrasound positive	104 (64.2)
	Sestamibi/SPECT-CT positive	86 (53.1)
	4D CT positive	49 (30.2)
	PET choline positive	11 (6.8)

Surgical findings and outcomes after reoperation

At reoperation, 71 (43.8%) underwent a focused or minimally invasive approach, 76 (46.9%) underwent bilateral exploration, and nine (5.6%) required sternotomy or thoracoscopic access. Intraoperative PTH monitoring was used in 73 (45.1%). The median number of glands removed was 1 (IQR: 1-2), and autotransplantation was performed in 22 (13.6%). Pathology revealed adenoma in 87 (53.7%), multigland hyperplasia in 54 (33.3%), carcinoma in six (3.7%), and other or non-diagnostic findings in 15 (9.3%). Postoperative complications occurred in 21 (13%), including permanent RLN injury in four (2.5%) and permanent hypoparathyroidism in six (3.7%). The median length of stay was three days (IQR: 2-5). At six months, 131 (80.9%) achieved biochemical cure. Eleven (6.8%) experienced late recurrence, and one (0.6%) died within 90 days of surgery (Table 4).

**Table 4:** Reoperation surgery and outcomes (n=162) Shown are the operative approaches, use of intraoperative PTH monitoring, pathology results, complications, and postoperative outcomes after reoperation. Cure at six months was defined as normocalcemia with the appropriate suppression of PTH on two or more occasions. PTH: parathyroid hormone; RLN: recurrent laryngeal nerve; IQR: interquartile range

Variable		Overall (n=162)
Approach, no. (%)	Focused/minimally invasive	71 (43.8)
	Bilateral exploration	76 (46.9)
	Sternotomy/thoracoscopic	9 (5.6)
	Other	6 (3.7)
Intraoperative PTH monitoring used, no. (%)		73 (45.1)
Number of glands removed, median (IQR)		1 (1-2)
Autotransplant performed, no. (%)		22 (13.6)
Pathology, no. (%)	Adenoma	87 (53.7)
	Hyperplasia	54 (33.3)
	Carcinoma	6 (3.7)
	Other/non-diagnostic	15 (9.3)
Complications, no. (%)	Any complication	21 (13)
	Transient RLN injury	7 (4.3)
	Permanent RLN injury	4 (2.5)
	Post-op hypocalcemia (transient)	12 (7.4)
	Post-op hypocalcemia (permanent)	6 (3.7)
Median length of stay (IQR), days		3 (2-5)
Cure at 6 months, no. (%)		131 (80.9)
Late recurrence after reoperation, no. (%)		11 (6.8)
90-day mortality, no. (%)		1 (0.6)

Outcomes according to indication for reoperation

Patients with recurrent disease had higher rates of cure compared with those with persistent disease, 89 of 103 (86.4%) versus 42 of 59 (71.2%) (p=0.02). Complication rates were not significantly different, 10 of 103 (9.7%) versus 11 of 59 (18.6%) (p=0.11). Permanent hypoparathyroidism occurred more frequently in the persistent group, five of 59 (8.5%) versus one of 103 (1%) (p=0.03). The median length of stay was longer in the persistent group, four days (IQR: 3-6) versus three days (IQR: 2-4) (p=0.04). Rates of late recurrence were not significantly different, six of 59 (10.2%) versus five of 103 (4.9%) (p=0.21) (Table 5).

**Table 5:** Outcomes by indication for reoperation (n=162) Shown are postoperative outcomes stratified by indication for reoperation (persistent vs. recurrent hyperparathyroidism). Continuous variables are expressed as median with IQR, and categorical variables as number (percentage). P-values are derived from the chi-squared test or Fisher's exact test for categorical variables and the Wilcoxon rank-sum test for continuous variables. IQR: interquartile range; LOS: length of stay; HPT: hyperparathyroidism

Outcome	Persistent (n=59)	Recurrent (n=103)	Test statistic	P-value
Cure at 6 months, no. (%)	42 (71.2)	89 (86.4)	χ²=5.27	0.02
Any complication, no. (%)	11 (18.6)	10 (9.7)	χ²=2.52	0.11
Permanent hypoparathyroidism, no. (%)	5 (8.5)	1 (1)	Fisher’s exact, χ²=4.74	0.03
Median LOS (IQR), days	4 (3-6)	3 (2-4)	z=2.05	0.04
Late recurrence, no. (%)	6 (10.2)	5 (4.9)	χ²=1.58	0.21

Predictors of cure

In multivariable logistic regression, recurrent disease (vs. persistent disease) was associated with a higher likelihood of cure at six months (adjusted odds ratio (OR): 2.4; 95% confidence interval (CI): 1.1-5.4; p=0.03). Positive localization on any imaging modality was also predictive of cure (adjusted OR: 3.1; 95% CI: 1.2-8.0; p=0.02). Adenoma pathology was independently associated with cure compared with hyperplasia (adjusted OR: 2.8; 95% CI: 1.2-6.4; p=0.01). Neither surgical approach nor intraoperative PTH monitoring reached statistical significance (Table 6).

**Table 6:** Multivariable predictors of cure at six months after reoperation (n=162) Shown are results from a logistic regression model assessing predictors of biochemical cure at six months. ORs are adjusted for disease type (persistent vs. recurrent), localization status, operative approach, pathology, and use of intraoperative PTH monitoring. PTH: parathyroid hormone; OR: odds ratio; CI: confidence interval; HPT: hyperparathyroidism

Variable	Adjusted OR (95% CI)	P-value
Recurrent (vs. persistent) HPT	2.4 (1.1-5.4)	0.03
Positive localization (any modality)	3.1 (1.2-8.0)	0.02
Focused/minimally invasive approach	1.6 (0.7-3.5)	0.23
Adenoma pathology (vs. hyperplasia)	2.8 (1.2-6.4)	0.01
Intraoperative PTH monitoring used	1.9 (0.9-4.1)	0.08

## Discussion

In this multicenter retrospective study from Saudi Arabia, it was found that reoperation for persistent or recurrent hyperparathyroidism resulted in a durable biochemical cure in approximately four of five patients. Cure was more likely in those with recurrent rather than persistent disease, in patients with positive preoperative localization, and in those whose pathology revealed adenoma rather than multiglandular hyperplasia. Despite the inherent challenges of reoperative neck surgery, complication rates were low, and the rates of permanent RLN injury and hypoparathyroidism were within the range reported in high-volume endocrine surgery series [2-4].

Our findings extend the existing literature in several respects [10-17]. Previous single-center studies have reported cure rates ranging from 70% to 85% after reoperation for persistent or recurrent disease, with lower success in persistent hyperparathyroidism, often due to missed multigland disease or ectopic adenomas [7,11]. In our cohort, cure was achieved in 86.4% of patients with recurrent disease and 71.2% of those with persistent disease, findings that align with this pattern. The strong predictive value of positive localization mirrors the experience reported with advanced imaging modalities, such as 4D CT and choline PET, which have increasingly been adopted to improve surgical planning in the reoperative setting [5-7]. Importantly, our data suggest that positive localization across any modality confers a threefold higher likelihood of cure, underscoring the central role of imaging in guiding reoperation.

The present results also inform surgical strategy. Focused approaches were feasible in nearly half of reoperations, particularly when reliable localization was available, and these were not associated with inferior outcomes compared with bilateral exploration. Adenoma pathology was independently associated with cure, highlighting the surgical curability of single-gland disease compared with multigland hyperplasia, which remains more challenging. Our complication rates for permanent RLN injury and permanent hypoparathyroidism were reassuringly low, despite the technical challenges posed by scarred and distorted operative fields.** **These outcomes support the practice of centralizing reoperations to experienced endocrine surgeons with access to intraoperative PTH monitoring and advanced imaging.

The implications for clinical practice are threefold. First, rigorous differentiation between persistent and recurrent disease is critical, as the prognosis differs substantially. Second, comprehensive preoperative localization should be pursued, ideally with multimodal imaging, to maximize cure and minimize morbidity. Third, patients should be counseled that although reoperation carries inherent risks, the likelihood of a durable cure is high when performed in specialized centers.

This study has limitations. Its retrospective design introduces the possibility of information bias and unmeasured confounding. Although data were collected systematically across participating centers, heterogeneity in imaging protocols, operative techniques, and follow-up schedules may have influenced outcomes. The sample size, while among the larger reported from the region, remains modest, and the number of patients with certain subgroups (e.g., carcinoma) was too small for definitive conclusions. Finally, although the median follow-up exceeded two years, late recurrence may have been underestimated. These limitations notwithstanding, the study provides robust, real-world evidence on the outcomes of reoperation for persistent and recurrent hyperparathyroidism in a Middle Eastern population.

## Conclusions

Reoperative parathyroid surgery can achieve favorable outcomes, with most patients attaining a cure when operations are guided by accurate preoperative localization and tailored surgical approaches. Our findings indicate that patients with recurrent disease are more likely to be cured than those with persistent disease and that positive localization studies and adenoma pathology further improve the likelihood of success. Although complications remain a concern, their overall rates are low, and permanent morbidity is uncommon. These results emphasize the importance of careful patient selection, optimal use of imaging, and experienced surgical management in maximizing cure while minimizing risk. Ultimately, reoperation offers a safe and effective treatment option for patients with persistent or recurrent hyperparathyroidism when performed under appropriate conditions.
